# Characterization of a GH8 β-1,4-Glucanase from *Bacillus subtilis* B111 and Its Saccharification Potential for Agricultural Straws

**DOI:** 10.4014/jmb.2105.05026

**Published:** 2021-08-20

**Authors:** Zhen Huang, Guorong Ni, Xiaoyan Zhao, Fei Wang, Mingren Qu

**Affiliations:** 1 Key Laboratory of Animal Nutrition of Jiangxi Province, Nutritional Feed Development Engineering Research Center, Jiangxi Agricultural University, Nanchang, Jiangxi 330045, P.R. China; 2 College of Land Resources and Environment, Jiangxi Agricultural University, Nanchang, Jiangxi 330045, P.R. China; 3 College of Bioscience and Bioengineering, Jiangxi Agricultural University, Nanchang, Jiangxi 330045, P.R. China

**Keywords:** *Bacillus subtilis*, endoglucanase, expression, characterization, oligosaccharide, saccharification

## Abstract

Herein, we cloned and expressed an endo-β-1,4-glucanase gene (*celA1805*) from *Bacillus subtilis* B111 in *Escherichia coli*. The recombinant celA1805 contains a glycosyl hydrolase (GH) family 8 domain and shared 76.8% identity with endo-1,4-β-glucanase from *Bacillus* sp. KSM-330. Results showed that the optimal pH and temperature of celA1805 were 6.0 and 50°C, respectively, and it was stable at pH 3-9 and temperature ≤50°C. Metal ions slightly affected enzyme activity, but chemical agents generally inhibited enzyme activity. Moreover, celA1805 showed a wide substrate specificity to CMC, barley β-glucan, lichenin, chitosan, PASC and avicel. The *K*_m_ and *V*_max_ values of celA1805 were 1.78 mg/ml and 50.09 μmol/min/mg. When incubated with cellooligosaccharides ranging from cellotriose to cellopentose, celA1805 mainly hydrolyzed cellotetrose (G4) and cellopentose (G5) to cellose (G2) and cellotriose (G3), but hardly hydrolyzed cellotriose. The concentrations of reducing sugars saccharified by celA1805 from wheat straw, rape straw, rice straw, peanut straw, and corn straw were increased by 0.21, 0.51, 0.26, 0.36, and 0.66 mg/ml, respectively. The results obtained in this study suggest potential applications of celA1805 in biomass saccharification.

## Introduction

Currently, the search for sustainable alternative energy sources has become a top priority due to the increased consumption and demand for fossil fuels. A previous study reported that the abundant cellulose biomass in agricultural straws can be used to produce biofuel ethanol, a potential alternative to fossil fuels [1]. Cellulose, the main backbone of plant biomass, is a liner polysaccharide consists of glucose units with β-1,4-bonds linkages [2, 3]. Cellulose degradation requires the synergistic action of multiple enzymes that are mainly classified into three groups. The first group is endo-β-1,4-glucanase (E.C. 3.2.1.4), which randomly cleaves the amorphous region of the polysaccharide chain to release small fragments. Subsequently, the fragments are hydrolyzed to cellobiose by exo-β-1,4-glucanase (E.C. 3.2.1.91) and finally to glucose by β-glucosidase (E.C. 3.2.1.21) [4, 5].

In recent years, endoglucanase has been the subject of widespread attention because of its wide application values in the biofuel, textile, and feed industries [5]. Endoglucanases can be classified into 12 glycosyl hydrolases families based on the similarity of catalytic domains of the amino acid sequence [6]. The key problems limiting industrial application of enzymes are how to reduce the production cost and improve the thermostability of enzymes. It has been reported that the GH8 family is an important part of many bacterial cellulases [7], and some GH8 endoglucanases have been identified from different bacteria and characterized. Studies have reported that these endoglucanases have broad substrate specificity, such as carboxy-methyl-cellulose (CMC), chitosan, barley-β-glucan, lichenin, and xylan [8, 9].

In our previous study, we isolated and identified a strain of *Bacillus subtilis* B111 from soil. Here, we cloned a cellulase gene (*celA1805*) from *B. subtilis* B111 into *Escherichia coli*, characterized its enzymatic properties, and determined its biomass saccharification rate.

## Materials and Methods

### Chemicals, Strains and Media

CMC, barley β-glucan (80% purity), filter paper, laminarin, corncobxylan, avicel, and chitosan were purchased from McLean (China), while lichenin was obtained from Megazyme (Ireland). Other unspecified chemicals were purchased from Sigma-Aldrich (USA).

*B. subtilis* B111 was used as the original gene cloning strain and was maintained in a lysogeny broth (LB). In addition, *E. coli* DH5α (Invitrogen Co., China) and *E. coli* BL21 (DE3) (Laboratory preservation) were used as hosts for heterologous expression, while the pET-29a plasmid (TakaRa, China) was used as the expression vector.

### Cloning of the celA1805 Gene

Genomic DNA was isolated from *B. subtilis* B111 and extracted using the method described by Kaiser [10], but with slight modification. PCR was performed to amplify the endoglucanase gene celA1805 (GenBank Accession No. MW248126) using the following primers: *celA1805*F (5′-TAAGAAGGAGATATACATATGTTGTATGTT ACATTTT ATGT-3′) and *celA1805*R (5′-GTGGTGGTGGTGGTGCTCGAGATTATCGTATC CTTCATA -3′). The PCR products were digested with NdeI and XhoI restriction enzymes, followed by linking into linearized pET29a (+) vector and addition of a 6× His-tag at the C-terminal. Transformants were then isolated and sequenced by Tsingke Corporation (China).

### Bioinformatics Analysis of Gene Sequences and Homologous Modeling

We predicted the isoelectric point and molecular mass using the expasy online tool (https://web.expasy.org). Next, we analyzed the protein sequence using the NCBI database BLASTP and conducted amino acid sequence alignment of celA1805 to other sequences using Clustal omega. Pfam database was then used to predict the structural domain and active site [9]. In addition, a phylogenetic tree was constructed using Mega 5.0 Software by applying the neighbor-joining method and Poisson model adjacency method. The 3D protein structure model of celA1805 was acquired using the SWISS-MODEL online server (https://swissmodel.expasy.org/interactive), with chitosanase from *Bacillus* sp. K17 (pdb: 1V5C) being used as the template since it shared 98.2% identity and 82%query cover of the celA1805 sequence. Finally, the crystal structure model was visualized using PyMol Molecular Graphics System.

### Expression and Purification of celA1805

We transformed the verified recombinant plasmid into *E. coli* BL21 (DE3) and cultured it in LB media (supplemented with kanamycin final concentration 50 μg/ml) at 37°C until the OD_600_ reached 0.5-0.6. For protein overexpression, 0.2 mM isopropyl-β-D-thiogalactopyranoside (IPTG) was induced at 16°C for 24 h. The recombinant strain cells were then centrifuged at 8,000 ×*g* for 20 min, followed by resuspension using 20 mM Tris-HCl at a pH of 7.0 and sonication. After centrifugation, the crude enzyme in the supernatant was purified with a Ni-NTA column, and eluted using Tris-HCl containing different concentrations of imidazole. The collected eluants were then analyzed using SDS-PAGE [11] and the protein concentration determined using the method described by Bradford [12].

### Properties of Recombinant celA1805

We measured the recombinant enzyme activity using 1% (w/v) CMC in 20 mM phosphate-buffered saline (PBS buffer) (pH 6.0) at 50°C for 1 h. On the other hand, the reducing sugar content was determined in accordance with the Dinitrosalicylic (DNS) method [13]. Notably, one unit of enzyme was defined as the amount of enzyme required to produce 1 μmol of glucose per minute.

Moreover, the optimal pH of celA1805 was assessed under standard condition (50°C, 1 h) in various 20 mM buffers (pH 3.0-10.5). We assessed pH stability by incubating aliquots of celA1805 in buffers ranging from pH 3.0 to10.5 for 24 h at 4°C, followed by determining the residual enzyme activity under standard conditions. We then determined the optimal reaction temperature ranging from 4°C to 70°C in PBS buffer (pH 6.0) for 1 h. Next, the effect of different temperatures on the stability of celA1805 was measured by incubating the solution in PBS buffer at 30-60°C up to 48 h. Meanwhile, the effects of various metal ions and chemicals was determined in PBS buffer (pH 6.0) with a 1 mM final concentration. Reactants without any additives were used as controls and enzyme activity was considered to be 100%. Finally, 1% (w/v) CMC, chitosan, barley β-glucan, lichenin, laminarin, avicel, phosphoric acid swollen cellulose (PASC), xylan, and filter paper were used to determine substrate specificity. It is worth noting that all experiments were performed in triplicate. In order to obtain the kinetic parameters of *K*_m_ and *V*_max_, enzyme activity was measured using various concentrations of CMC (1.0-8.0 mg/ml) under optimal conditions.

### Analysis of Hydrolytic Cello-Oligosaccharides Products

To confirm the mode of celA1805 hydrolysis, we added 1 mg/ml of cello-oligosaccharides (G2-G5) in sodium phosphate buffer (pH 6.0) with 0.1 U recombinant enzyme at 50°C. Samples were withdrawn after 6 h and 12 h, and boiled for 10 min. Next, the end products were analyzed using a silicone glass plate (Merck, Germany). The end products were developed using n-butanol: water: acetic acid (2:1:1 v/v/v) solvent [14]. Products were then visualized by spraying in sulfuric/methanol acid (1:4, v/v) solution, followed by heating at 100°C until they became visible.

### Saccharification of Agricultural Straws

We crushed different agricultural straws (1% (w/v)) through a 40-mesh sieve and blended them in 20 mM PBS buffer with 0 or 0.45 U crude recombinant enzyme. The mixture was loaded into a 10 ml centrifuge tube and incubated on a rotary shaker (150 rpm/min) for 48 h at 50°C. We then boiled the mixture for 10 min, followed by centrifugation at 10,000 ×*g* for 20 min. Finally, we assessed the released reducing sugars in the supernatants using the DNS method, and subsequently calculated the saccharification rate [15].

## Results and Discussion

### Analysis of celA1805 Sequence and Homologous Modeling

**Fig. 1:**
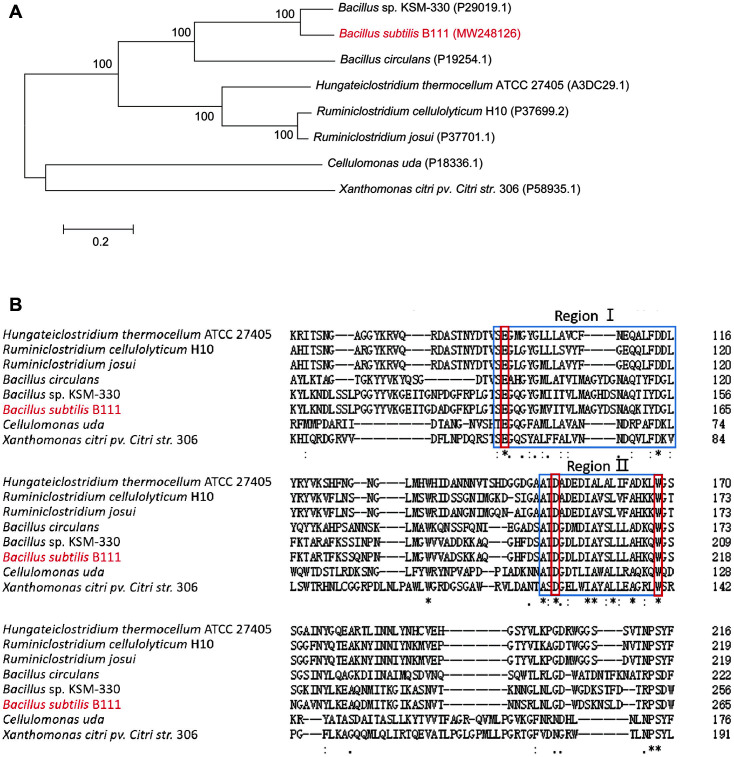
(**A**) A phylogenetic tree of celA1805 and related β-1,4-glucanases from different sources. (**B**) Alignment of amino acid sequence between celA1805 and other related β-1,4-glucanases. The blue boxes represent conserved amino acid sequences, while red boxes represent active sites.

Sequence analysis revealed the existence of a 1,413 bp open reading frame (ORF), which encodes 470 amino acids without a signal peptide. The predicted molecular weight was 52.4 kDa and the theoretical pI was 8.5. In addition, the phylogenic tree (Fig. 1A) showed that celA1805 was on the same branch as bglS from *Bacillus* sp. KSM-330 (NCBI, Accession No. P29019.1). BLAST analysis showed that celA1805 had a 76.8% homology with endo-1,4-β-glucanase from *Bacillus* sp. KSM-330 [16], followed by 43.4% homology with endo-β-1,3-1,4-glucanase (P19254.1) from *Bacillus circulans* [17]. It also shared about 30% sequence identity with endo-1,4-beta-glucanases from other bacteria in the GenBank database.

Prediction of celA1805 using Pfam database revealed the presence of a GH8 family structural domain. Multiple alignment analysis of amino acid sequences showed two conserved sequences, with amino acid sequences in one region ranging from 138 to 165 (region I) and the other one ranging from 198 to 216 (region II) (Fig. 1B). The second region was found to be present in many GH8 family menbers [9]. Previous studies have reported that glutamate and aspartic acid are highly conserved amino acid residues necessary for GH8 enzyme catalytic activity [18], and Glu95 (in region I) and Asp156 (in region II) are active sites of the endo-β-1,3-1,4-glucanase from *Bacillus circulans* [17]. Moreover, at least one Trp residue in region II was be involved in enzymatic catalysis [19].

**Fig. 2:**
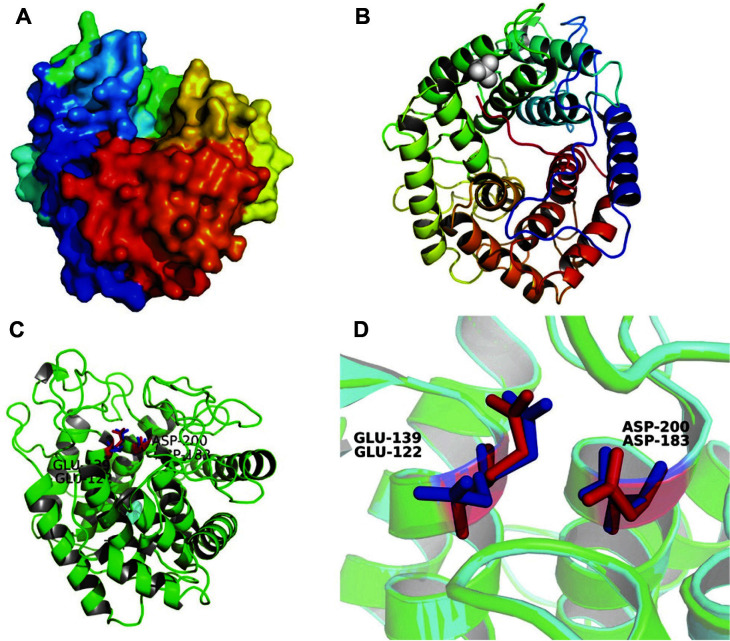
3D structure model of celA1805 protein. (**A**) Surface display of predicted structure. (**B**) Ribbon display of 3D structure. (**C**) The active site within the cleft of the protein. Green ribbon represents celA1805 and cyan ribbon represents 1V5C. (**D**) Amino acid residue structure of the active site. Red sticks represent the amino residues of celA1805 and blue sticks represent the amino residues of 1V5C.

Our results showed that celA1805 is a round protein molecule with a cavity in the middle (Fig. 2A), within which the active center amino acid residues are located. In addition, celA1805 has a typical two-layer (α/α) 6-barrel structure (Fig. 2B) of the glycoside hydrolase GH8 family, which includes chitosanase (E.C. 3.2.1.132) [20], cellulase (E.C. 3.2.1.4) [21], licheninase (E.C. 3.2.1.73) [22], endo-1,4-β-xylanase (E.C. 3.2.1.8) [23], and reducing-end-xylose releasing exo-oligoxylanase (E.C. 3.2.1.156) [24]. Similar to 1V5C (cyan ribbon), the amino acid residues of the active center of celA1805 (green ribbon) consisted of Glu139 and Asp200 (red sticks) (Fig. 2C), with Asp200 acting as the catalytic nucleophile and Glu139 acting as a proton donor (Fig. 2D) [16, 20].

### Expression and Purification of celA1805

**Fig. 3:**
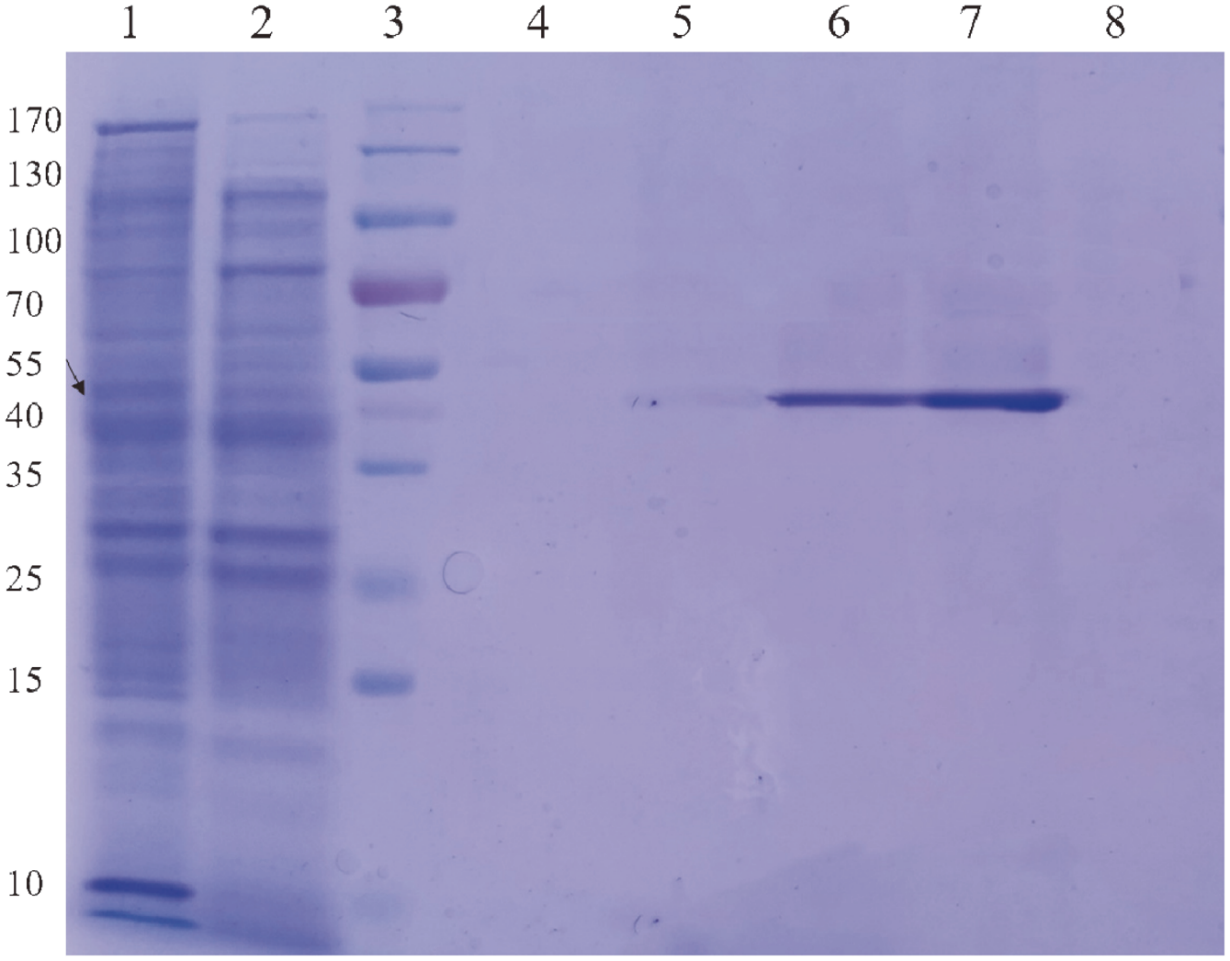
Expression and purification of celA1805. Lane **1**: crude enzyme from the cell lysates supernatant of recombinant strain; lane **2**: proteins eluted with 0 mM imidazole; lane **3**: protein marker; lane **4**: proteins eluted with 50 mM imidazole; lane **5**: proteins eluted with 100 mM imidazole; lane **6**: proteins eluted with 200 mM imidazole; lane **7**: proteins eluted with 300 mM imidazole; lane **8**: proteins eluted with 400 mM imidazole.

There was soluble expression of the *celA1805* gene in *E. coli* and the recombinant celA1805 induced by IPTG was present in the supernatant (Fig. 3A). Results showed that the recombinant celA1805 was successfully purified through Ni-NTA affinity chromatography (Fig. 3), with specific enzyme activity and CMC being used as the substrate. In addition, the molecular mass analyzed from SDS-PAGE (53 KDa) was consistent with the estimated value (52.4 KDa). This indicated that the molecular weight of celA1805 is larger than that of other GH8 family endoglucanases from mesophilic bacteria, such as GH8ErCel (38 KDa) from *Enterobacter* sp. [8], BGlc8H (40 KDa) from *Paenibacillus* sp. X4 [9], Cel8H (36 KDa) from *Halomonas* sp. S66-4 [25], and Cel8A (39 KDa) from *Serratia proteamaculans* CDBB-1961 [26]. However, the molecular weight of celA1805 is similar to Cel8A (52KDa) from thermophilic anaerobic bacterium *Clostridium themocellum* [27].

### Characteristics of Recombinant celA1805

**Fig. 4:**
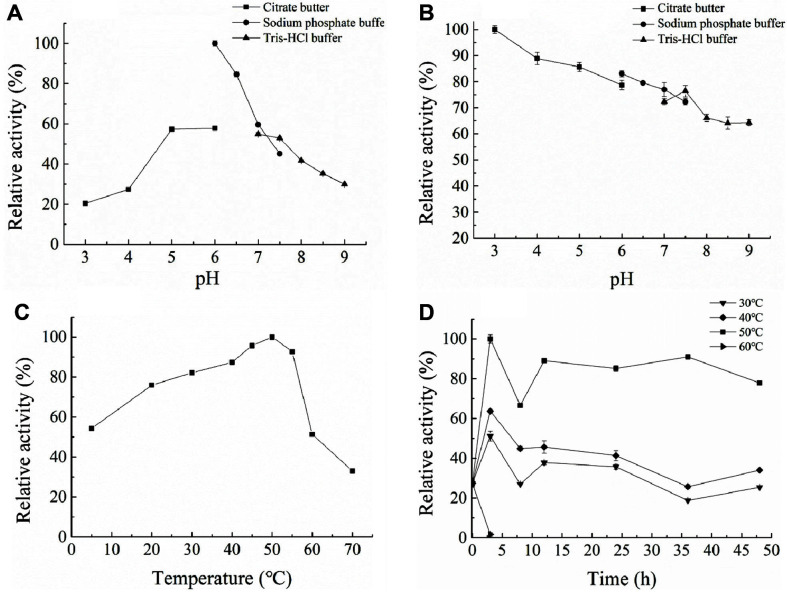
Optimum pH and temperature, and stability of celA1805. (**A**) Optimal pH. The optimum pH was determined at 50°C for 1 h, in various pH buffers. (**B**) pH stability. Residual activity was assessed under optimum conditions (20 mM PBS buffer, pH 6.0, 50°C, 1 h) after mixing the purified celA1805 with different buffers at 4°C for 24 h. (**C**) Optimum temperature. Optimum activity was examined in 20 mM PBS (pH 6.0) at temperatures ranging from 4 to 70°C for 1 h. (**D**) Thermostability. Enzyme activity was assessed by incubating in 20 mM PBS (pH 6.0) at 30-60°C for 48 h.

Fig. 4A shows that the optimum pH of recombinant celA1805 was 6.0. It exerted 60% activity at pH 5 and 7, and more than 20% activity at pH 3 and 9. The optimum pH of celA1805 was slightly higher than Cel9K (pH 5.5) [28] from *Paenibacillus* sp. X4 and *Bacillus* sp. KSM-330 [19], and lower than GH8ErCel (pH 7.0) from *Enterobacter* sp. R1 [8]. When the enzyme was placed in different buffers for 24 h, it showed strong stability to acid and the most stable pH was 3.0, while residual enzyme activity remained 60% at pH 9.0 (Fig. 4B). The pH stability results are different from that of Bacillus sp. KSM-330 (pH 5.2). Generally, most of the reported enzymes are highly active at acidic to neutral pH [15, 29, 30]. Moreover, the optimum temperature was 50°C (Fig. 4C), which was similar to BGlc8H from *Paenibacillus* sp. X4 [9], but slightly lower than Egl-257 from *Bacillus circulans* KSM-N257 (55°C)[31] and Pgl8A from *P. cookii* (55°C) [18]. Notably, celA1805 retained >80% activity after 48 h treatment at 50°C, but the activity dropped to about 30% at 30°C or 40°C (Fig. 4D). However, it was still much more stable than the activity of Cel9K and Egl-257 [28]. In addition to celA805, there are a few endoglucanases with thermostability. For example, the residual activity of Cen219 from *Enterobacter* sp. was 80% after incubation at 50°C for 24 h [32], and GH8ErCel from *Enterobacter* sp. retained 50% residual activity after 48 h at 60°C [32]. However, the enzyme activity increased rapidly after 48 h heat treatment, especially at 50°C. Similar results were obtained on fused enzymes from C. saccharolyticus [33]. Collectively, our results suggested that the enzyme activity may be stimulated at a certain high temperature and was stable at 50°C, which is beneficial for enzyme stability after heat treatment and reducing enzyme storage costs.

**Fig. 5:**
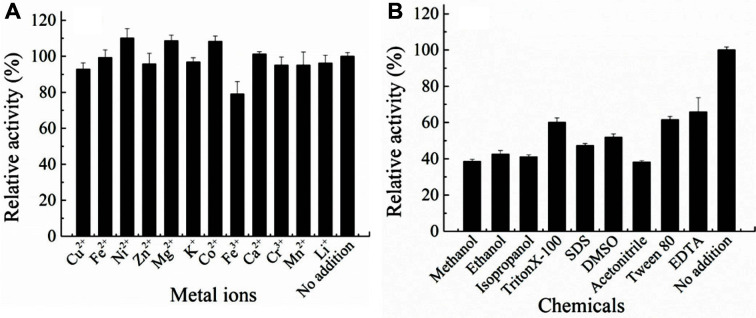
Effect of (**A**) metal ions and (**B**) chemicals on the activity of celA1805.

Fig. 5A shows that the metal ions did not significantly influence the celA1805 activity. Similar results were reported with *Bacillus* sp. KSM-330 [16]. Only 1 mM Ni^2+^, Mg^2+^, and Co^2+^ increased the enzyme activity to 111.2%, 108.7%, and 108.3%, respectively. On the other hand, Fe^3+^ inhibited the enzyme activity to 79.1%, while the other metal ions slightly inhibited the activity. However, most of the chemicals inhibited the residual enzyme activity of celA1805 to less than 50%, including 10% (v/v) methanol, ethanol, isopropanol, acetonitrile, and 2 mg/ml SDS. It is worth noting that the slight inhibition by metal ions is beneficial for widespread industrial application of celA1805.

### Substrate Specificity of celA1805

As shown in Table 1, celA1805 exerted high activity towards CMC (7.66 U/mg), barley β-glucan (12.87 U/mg), lichenin (3.72 U/mg), chitosan (13.49 U/mg) and PASC (3.41 U/mg), but low activity towards filter paper (0.78 U/mg) and avicel (0.46 U/mg). This indicated that celA1805 can hydrolyze β-1,4-glucan linkages and β-1,3-1,4-glucan linkages, but it cannot hydrolyze β-1,3-glucan linkages. The higher activity towards barley β-glucan than lichenin can be attributed to the higher proportion of β-1,4-linkages to β-1,3-linkages in barley β-glucan [34]. However, celA1805 was refined as endoglucanase and not lichenase according to the Nomenclature Committee of the International Union of Biochemistry and Molecular Biology (IUBMB), because lichenase hydrolyzes cereal mixed β-1,3-1,4-linkages but not pure β-1,4-linkages. Endo-K from *Bacillus* sp. KSM-330, which shared the highest amino acid sequence homology to celA1805, showed high activity to CMC (100%) and lichenin (115.6%), but barely hydrolyzed avicel, PASC, curdlan, and laminarin [19]. In this study, celA1805 showed higher activity to PASC compared to Endo-K. Studies have shown that the GH8 family includes chitosanase, cellulose, lichenase, and xylanase [35, 36]. Unlike other GH8 endoglucanases, celA1805 exerted substrate specificity more widely than GH8ErCel from *Enterobacter* sp. R1 (Table 2) [8], CEL8M from Ladakh soil by functional metagenomics [37], Cel8A from *Lysobacter* sp. IB-9374 [38], Egl-257 from *Bacillus circulans* [31], Cel8H from *Halomonas* sp. S66-4 [25], and Cel8A from *Serratia proteamaculans* CDBB-1961 [26], but showed no xylanase activity [9, 37].

The kinetic parameters of celA1805 were plotted using the method of Lineweaver and Burk. The *K*_m_ and *V*_max_ of celA1805 with CMC substrate were 1.78 mg/ml and 50.09 μmol/min/mg. The values of endoglucanases *K*_m_ have been reported to range from 0.01 to 6.6 mg/ml toward CMC [39, 40]. In this study, the *K*_m_ was lower than most reports of GH8 endoglucanases, such as Cel8A (6.6 mg/ml) from *Serratia proteamaculans* CDBB-1961 [26], Cel8H (37.5 mg/ml) from *Halomonas* sp. S66-4 [25], Egl-257 (7.1 g/l) from *Bacillus circulans* [31], CEL8M (10 mg/ml) from Ladakh soil by functional metagenomics [37] and Cen219 (17.37 mg/ml) from a *Bursaphelenchus xylophilus* metagenomic library [32]. The *V*_max_ of celA1805 was higher than Cel8A (3.5 μmol/min/mg) but lower than Cen219 (333.33 U/mg).

### Analysis of Hydrolytic Cello-Oligosaccharides Products

**Fig. 6:**
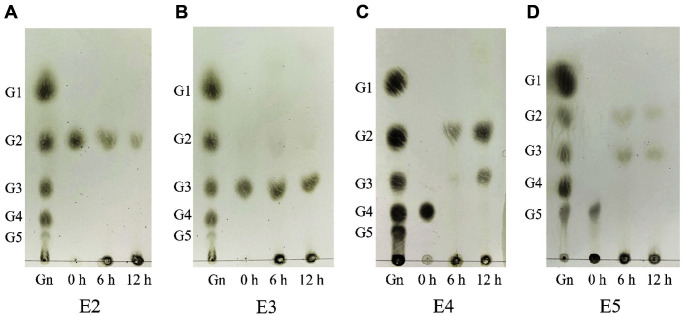
TLC analysis of the celA1805 hydrolysis products from cello-oligosaccharides (G_2_-G_5_) for 6 h and 12 h respectively. 200 μl cello-oligosaccharides (1 mg/ml) with 0.1 U celA1805 added into 20 mM PBS buffer (pH 6.0) and incubated at 50°C for 12 h, after which samples were loaded and analyzed through TLC. (**A**), (**B**), (**C**), and (**D**) represented celA1805 hydrolysis products of reaction with cellobiose, cellotriose, cellotetraose and cellopentose respectively. (Gn): Standard substances, including glucose (G1), cellobiose (G2), cellotriose (G3), cellotetraose (G4) and cellopentose (G5). E2, E3, E4, and E5 were enzyme reaction samples with G2, G3, G4, and G5 respectively.

To determine the mode of action of celA1805, we used TLC to analyze the hydrolyzed products in the cello-oligosaccharides reaction (Figs. 6A and 6B). Results showed that celA1805 hydrolyzed G5 rapidly at 6 h, had relatively slow hydrolysis of G4 after 12 h, and did not hydrolyze G3 in the reaction condition. In addition, celA1805 hydrolyzed G5 to G2, and G3 and G4 to G2 and G3. These results suggest that celA1805 is an endo-type β-1,4-glucanase because its modes of hydrolysis and hydrolysates are similar to those of endo-β-1,4-glucanase, such as Cel6H-p35 from *Eisenia fetida* and EF-EG2 from a compost metagenomic library [41, 42].

### Saccharification of Agricultural Straws

Furthermore, we used agricultural straws to verify the saccharification ability of celA1805. Our results indicated that the reducing sugars released in the celA1805 treatment groups were significantly increased compared to the control group after incubation for 48 h. Moreover, celA1805 promoted enzymatic hydrolysis of all agricultural straws. The increased amounts for wheat straw, rape straw, rice straw, peanut straw, and corn straw were 0.21, 0.51, 0.26, 0.36, and 0.66 mg/ml, respectively. The different enzymolysis efficiency to agricultural straws can be attributed to the difference in lignocellulose structure and contents. Our results were similar to a previous report that Cel-5A hydrolyzed non-pretreated biomass materials [43]. Collectively, these findings suggest that celA1805 has potential industrial application as a biomass pretreatment enzyme in ethanol production.

In conclusion, a new GH8 endoglucanase gene from *B. subtilis* B111 was cloned and characterized. celA1805 exerted wide substrate specificity, with distinctive activity towards various glucans containing β-1,4-linkages. In addition, celA1805 exhibited broad pH stability and thermostability, and is therefore expected to be a potential enzyme for the feed industry as well as for biomass saccharification to produce ethanol.

## Figures and Tables

**Fig. 7:**
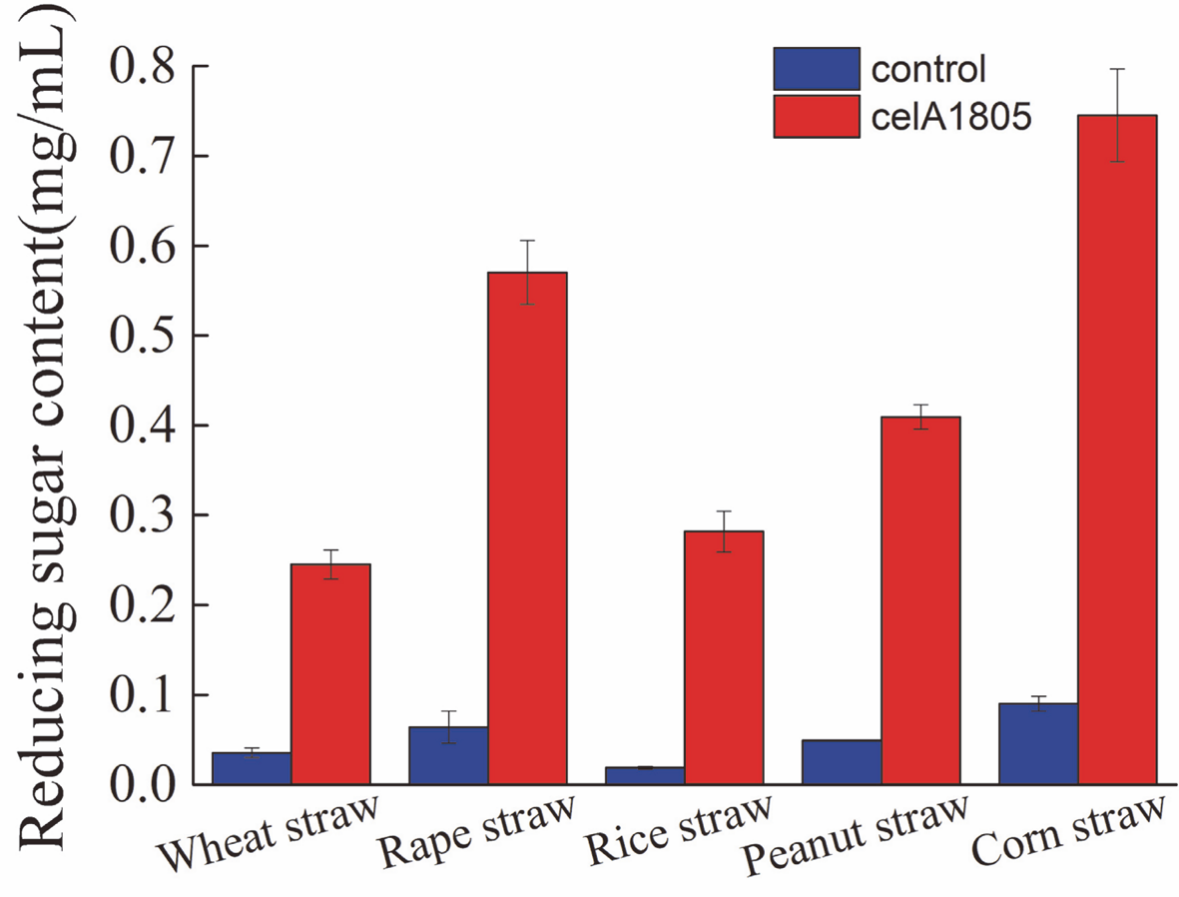
Yield of reducing sugars released from agricultural straws after hydrolysis by celA1805. Recombinant celA1805 (0 or 0.45 U/g straws) was added in 1% (W/V) agricultural straws incubated in 20 mM PBS (pH 6.0) buffer with shaking (150 r/min) at 50°C for 48 h. Concentration of reducing sugars was verified through the DNS method. In addition, the reaction mixtures were boiled for 10 min.

**Table 1:** Substrate specificity of celA1805.

Substrate	Specific Activity (U/mg)
CMC	7.66±0.48
Chitosan	13.49±0.13
Barley β-glucan	12.87±0.22
Lichenan	3.72±0.07
Filter paper	0.78±0.02
PASC	3.41±0.03
Avicel	0.46±0.05

**Table 2:** Comparison of enzyme properties of celA1805 and other GH8 β-1,4-glucanases.

Enzyme	Source	Molecular size (KDa)	Optimal pH	Optimal temperature	pH stability	Thermal stability	Specific enzyme activity(U/mg)							Reference

							CMC	Barley β-glucan	Lichenin	Chitosan	PASC	Xylan	Avicel	
CelA1805	*Bacillus subtilis* B111	52.4	6.0	50°C	3-9	4-60°C	7.66	12.87	3.72	13.49	3.41	-	0.46	This study
GH8ErCel	*Enterobacter* sp. R1	38	7	60°C	5-7	20-60°C	4.1	49.8	60.6	-	1.9	-	-	[44]
Cel8Pa	*Paenibacillus xylanivorans* A59	-	4.5	40°C	-	5-20°C	7.35	24.52	17.87	1.38	9.73	0.40	-	[45]
Bglc8H	*Paenibacillus* sp. X4	41.6	5	50°C	-	-	0.71	2.22	0.32	1.80	-	0.02	-	[46]
CEL8M	Ladakh soil by functional metagenomics	38.9	4.5	28°C	-	10-40°C	4.75	0.62	-	-	-	-	-	[37]
Pgl8A	*Paenibacillus cookie*	41.8	5.1	-	-	-	50.9	-	13.4	149	-	-	-	[18]
Cel8A	*Lysobacter* sp. IB-9374	41	-	-	5-8	4-40°C	484	-	-	128	-	-	-	[38]
celA	*Clostridium thermocellum*	52	5.5-6.5	75°C	-	<60°C	580	-	2910	2204	208	-	-	[27]
Egl-257	Bacillus circulans	43	8.5	55°C	5-11	<55°C -	100%^a^	-	41.7%^b^	-	-	-	-	[31]
Cel8H	*Halomonas* sp. S66-4	36	5	45°C	4-12	40-60°C	4.9	-	-	-	-	-	-	[25]
Cen219	*Bursaphelenchus xylophilus*	40	6	50°C	4-7	30-50°C	107.24	189.63	82.44	-	-	-	26.8	[32]
Cel8A	*Serratia proteamaculans* CDBB-1961	41.2	7	40°C	4-8.5	15-50°C	0.85	-	-	-	-	c	-	[26]

-Not available.

^a,b^Relative enzyme activity.

^c^Lack of specific dada.
